# Almost Sure GOE Fluctuations of Energy Levels for Hyperbolic Surfaces of High Genus

**DOI:** 10.1007/s00023-025-01552-4

**Published:** 2025-02-22

**Authors:** Zeév Rudnick, Igor Wigman

**Affiliations:** 1 School of Mathematical Sciences, Tel Aviv University, 69978 Tel Aviv, Israel; 2 Department of Mathematics, King’s College London, London, UK

## Abstract

We study the variance of a linear statistic of the Laplace eigenvalues on a hyperbolic surface, when the surface varies over the moduli space of all surfaces of fixed genus, sampled at random according to the Weil–Petersson measure. The ensemble variance of the linear statistic was recently shown to coincide with that of the corresponding statistic in the Gaussian orthogonal ensemble (GOE) of random matrix theory, in the double limit of first taking large genus and then shrinking size of the energy window. In this note, we show that in this same limit, the (smooth) energy variance for a typical surface is close to the GOE result, a feature called “ergodicity” in the random matrix theory literature.

## Introduction

### Statement of Results

Let *X* be a compact hyperbolic surface of genus $g\ge 2$, $\lambda _{j}=1/4+r_{j}^{2}$ the Laplace eigenvalues on *X*. Let *f* be a test function so that its Fourier transform ${\widehat{f}}$ is even, smooth and compactly supported, $L\ge 1$, $\tau \in {{\mathbb {R}}}$, and define the smooth linear statistic

1.1 $$\begin{aligned} N_{L,\tau }(X) := \sum \limits _{j\ge 0}f(L(r_{j}-\tau ))+f(L(r_{j}+\tau )) , \end{aligned}$$

effectively counting eigenvalues in a window of size $\approx \tau /L$ around $\tau ^2$ (for $\tau >0$), as in [7, 10–12]. Here we assume, by convention, that $r_{j}\in {{\mathbb {R}}}_{\ge 0}\cup i{{\mathbb {R}}}_{\ge 0}$. We write $N_{f,L,\tau }(X)$ as a sum of a smooth and fluctuating terms

$$\begin{aligned} N_{L,\tau }(X) = {\overline{N}} + {\widetilde{N}}_{L,\tau }(X) , \end{aligned}$$

where

$$\begin{aligned} {\overline{N}}:= 2(g-1)\int \limits _{-\infty }^{\infty }f(L(r-\tau ))r\tanh (\pi r)dr, \end{aligned}$$

and it can be shown [11, Sect.3] that, for $L>1$ fixed, as $\tau \rightarrow \infty$,

$$\begin{aligned} {\overline{N}} \sim \frac{2\tau (g-1)}{L}\int \limits _{-\infty }^{\infty }f(x)dx, \end{aligned}$$

provided that

$$\begin{aligned} \int \limits _{-\infty }^{\infty }f(x)dx\ne 0. \end{aligned}$$

In [11], it was shown that when averaged over the moduli space ${\mathcal {M}}_g$ of surfaces of genus *g* with respect to the Weil–Petersson measure, the variance of ${\widetilde{N}}_{L,\tau }(X)$ matches that of the corresponding statistic in the Gaussian orthogonal ensemble (GOE) of random matrix theory, in the double limit, taking $g\rightarrow \infty$, and then $L\rightarrow \infty$ (with $\tau$ fixed):

1.2 $$\begin{aligned} \lim _{L\rightarrow \infty } \lim _{g\rightarrow \infty }{\mathbb {E}}^{\textrm{WP}}_g\left( \left| {\widetilde{N}}_{L,\tau } -{\mathbb {E}}^{\textrm{WP}}_g( {\widetilde{N}}_{L,\tau }) \right| ^2 \right) = \Sigma ^2_{\textrm{GOE}}(f) \end{aligned}$$

where $$\Sigma ^2_{\textrm{GOE}}(f) = 2\int _{-\infty }^\infty |x|{\widehat{f}}(x)^2dx$$.

We now consider the energy average of $N_{L,\tau }(X)$ (or ${\widetilde{N}}_{L,\tau }(X)$). Let $\omega$ be a nonnegative, even weight function, normalized by $\int _{-\infty }^\infty \omega (x)dx=1$, with Fourier transform ${\widehat{\omega }}$ smooth and supported in $[-1,1]$. For $T>0$, define an averaging operator

1.3 $$\begin{aligned} {{\mathbb {E}}}_T[F]:=\frac{1}{T}\int _{-\infty }^\infty F(\tau )\omega \left( \frac{\tau }{T} \right) d\tau \end{aligned}$$

and a corresponding variance

1.4 $$\begin{aligned} \operatorname {Var}_T(F)= {{\mathbb {E}}}_T\left[ \left| F-{{\mathbb {E}}}_T[F] \right| ^2 \right] . \end{aligned}$$

Denote by ${\mathcal {V}}_{T,L}(X)$, the energy variance of ${\widetilde{N}}_{L,\tau }(X)$, thought of as a random variable on ${\mathcal {M}}_g$:

$$\begin{aligned} {\mathcal {V}}_{T,L}(X) :=\operatorname {Var}_{T}({\widetilde{N}}_{L,\tau }(X)) . \end{aligned}$$

Following Berry [1] (see [1, Equations (23)-(24) on p. 52] for the energy fluctuations, without smoothing), it is believed that for *generic* surfaces^1^$X\in {\mathcal {M}}_g$, for fixed genus^2^$g>2$, the energy variance ${\mathcal {V}}_{T,L}(X)$ will converge to the GOE variance $\Sigma ^2_{\textrm{GOE}}(f)$ when $T\rightarrow \infty$, and $L \rightarrow \infty$ but $L=o(T)$. Our principal result supports this if we first take the large genus limit $g\rightarrow \infty$, and then the high energy limit $T\rightarrow \infty$, while restricting to energy windows $L\rightarrow \infty$ with $L=o(\log T)$, more precisely that in this limit the random variable ${\mathcal {V}}_{T,L}(X)$ converges in distribution to the constant $\Sigma ^2_{\textrm{GOE}}(f)$:

#### Theorem 1.1

For every $$\epsilon >0$$,

1.5 $$\begin{aligned} \lim \limits _{\begin{array}{c} L,T\rightarrow \infty \\ L=o(\log {T}) \end{array}}\limsup \limits _{g\rightarrow \infty } \operatorname {Prob}^{WP}_{g}\left( \left| {\mathcal {V}}_{T,L} - \Sigma _{GOE}^{2}(f) \right| > \epsilon \right) = 0 . \end{aligned}$$

This may be viewed as an analogue of a feature called “ergodicity” in random matrix theory: In the limit of large matrix size, the energy variance equals the ensemble variance for almost all matrices, see [9].

### Method of Proof

Using the Selberg trace formula, we have [11]

$$\begin{aligned} N_{L,\tau }(X)= {\overline{N}} +{\widetilde{N}}_{L,\tau }(X) \end{aligned}$$

with

$$\begin{aligned} {\overline{N}} =2(g-1)\int \limits _{-\infty }^{\infty }f(L(r-\tau ))r\tanh (\pi r)dr\sim \frac{2\tau (g-1)}{L}\int \limits _{-\infty }^{\infty }f(x)dx \end{aligned}$$

(asymptotic holding as $\tau \rightarrow \infty$, see above), and

1.6 $$\begin{aligned} {\widetilde{N}}_{L,\tau }(X) =\frac{2}{L}\sum \limits _{\ell _\gamma \in {\mathcal {L}}_g(X) }\sum \limits _{k\ge 1}\frac{\ell _{\gamma }}{\sinh (k\ell _{\gamma }/2)}{\widehat{f}}\left( \frac{k\ell _{\gamma }}{L}\right) \cos (\tau k\ell _{\gamma }) \end{aligned}$$

where the summation is over the primitive length spectrum

$$\begin{aligned} {\mathcal {L}}_g(X)\subset (0,\infty ), \end{aligned}$$

the set of lengths of primitive non-oriented closed geodesics of *X* (with possible multiplicities).

We view the primitive length spectrum as a random point set ${\mathcal {L}}_{g}$, parameterized by the random variable $X\in {\mathcal {M}}_g$, that is a random point process, and the variance ${\mathcal {V}}_{T,L}(X)$ is an application $\varphi ({\mathcal {L}}_{g})$ of a functional $\varphi$ on ${\mathcal {L}}_{g}$, which is continuous in a suitable topology. A fundamental result of Mirzakhani and Petri [8] shows that the point processes ${\mathcal {L}}_g$ converge, in distribution as $g\rightarrow \infty$, to a Poisson point process, denoted ${\mathcal {L}}_{\infty }$ on the positive reals, with intensity measure

$$\begin{aligned} d\nu _{MP}(t) = \frac{2\sinh (t/2)^{2}}{t} dt. \end{aligned}$$

From the theory of point processes, it follows that ${\mathcal {V}}_{T,L}(X)=\varphi ({\mathcal {L}}_{g})$ converges, in distribution as $g\rightarrow \infty$ to the random variable ${\mathcal {V}}_{T,L}^\infty = \varphi ({\mathcal {L}}_{\infty })$, see Proposition 2.2. To show Theorem 1.1, we argue that it suffices to prove that ${\mathcal {V}}_{T,L}^\infty$, converges in distribution to the constant $\Sigma ^2_{\textrm{GOE}}(f)$ as $L,T\rightarrow \infty$ with $L=o(\log T)$ (see Theorem 2.3). The proof of the latter result takes up the bulk of this paper and is done in Sect. 4.

## Outline of the Proof of the Main Result

We rewrite (1.6) as

2.1 $$\begin{aligned} {\widetilde{N}}_{L,\tau }(X) =\sum _{\ell \in {\mathcal {L}}_{g}} H_{L,\tau }(\ell ), \end{aligned}$$

where

2.2 $$\begin{aligned} H_{L,\tau }(x)= 2\frac{x}{L} \sum _{k\ge 1} \frac{{\widehat{f}}(\frac{kx}{L}) \cos (kx \tau )}{\sinh (kx/2)} \end{aligned}$$

that we think of as a functional of the length spectrum, that is considered as a random point process, as in the following notation.

### Notation 2.1

1. For $g\ge 1$, let ${\mathcal {L}}_{g}={\mathcal {L}}_{g}(X)=\{\ell _{j}\}_{j\ge 1}$ be the (random) primitive unoriented length spectrum of $X\in {\mathcal {M}}_g$, considered as a random point process on ${{\mathbb {R}}}_{>0}$.
2. Let ${\mathcal {L}}_{\infty } = \{\ell _{j}\}_{j\ge 1}$ be the Poisson point process on ${{\mathbb {R}}}_{>0}$ of intensity
  2.3 $$\begin{aligned} \nu _{MP}(dt):=\frac{2\sinh (t/2)^{2}}{t}dt. \end{aligned}$$

It was proved by Mirzakhani–Petri [8], that ${\mathcal {L}}_{g}$ converges, as a sequence of point processes, to ${\mathcal {L}}_{\infty }$, see Sect. 3. It is then natural to consider the analogue of ${\widetilde{N}}_{L,\tau }(X)$, with ${\mathcal {L}}_{g}$ replaced by ${\mathcal {L}}_{\infty }$. That is, define the random variable

2.4 $$\begin{aligned} S_{L,\tau } = \sum _{\ell \in {\mathcal {L}}_{\infty }} H_{L,\tau }(\ell ), \end{aligned}$$

where $H_{L,\tau }(x)$ is given by (2.2). We recall the averaging operator ${{\mathbb {E}}}_{T}[\cdot ]$, as in (1.3), and apply the corresponding variance operator $\operatorname {Var}_T(\cdot )$ as in (1.4), on $S_{L,\tau }$:

$$\begin{aligned} {\mathcal {V}}_{T,L}^{\infty }:=\operatorname {Var}_{T}(S_{L,\tau })={{\mathbb {E}}}_T\left[ \left| S_{L,\tau }(X)-{{\mathbb {E}}}_T\left[ S_{L,\tau }\left( X\right) \right] \right| ^2\right] . \end{aligned}$$

We aim to express ${\mathcal {V}}_{T,L}^{\infty }$ directly in terms of ${\mathcal {L}}_{\infty }$.

To this end, squaring (2.4), after some simple manipulations with the resulting expression, gives

2.5 $$\begin{aligned} {\mathcal {V}}_{T,L}^{\infty } = \left( \frac{2}{L} \right) ^2\sum _{\ell _1,\ell _2\in {\mathcal {L}}_{\infty }} \sum _{k_1,k_2\ge 1} \frac{ H_L(k_1\ell _1) H_L(k_2\ell _2) }{k_1 k_2 } U_T(k_1\ell _1,k_2\ell _2) \end{aligned}$$

with

2.6 $$\begin{aligned} H_L(x) = \frac{x{\widehat{f}}\left( \frac{x}{L} \right) }{\sinh (x/2)} \end{aligned}$$

and

2.7 $$\begin{aligned} \begin{aligned} U_T(x,y)&={{\mathbb {E}}}_T \left[ \cos \left( \tau x\right) \cos \left( \tau y\right) \right] -{{\mathbb {E}}}_T \left[ \cos \left( \tau x \right) \right] \cdot {{\mathbb {E}}}_T \left[ \cos \left( \tau y\right) \right] \\&= \frac{1}{2} \widehat{\omega }\left( T\left( x-y\right) \right) + \frac{1}{2} {\widehat{\omega }}\left( T\left( x+y\right) \right) -{\widehat{\omega }}\left( Tx\right) \cdot {\widehat{\omega }}\left( Ty\right) . \end{aligned} \end{aligned}$$

Note that $U_T(x,y)$ is real-valued since $\omega (x)$ is real-valued and even. Starting from (2.1) in place of (2.4), and likewise yields

2.8 $$\begin{aligned} {\mathcal {V}}_{T,L}(X) = \left( \frac{2}{L} \right) ^2\sum _{\ell _1,\ell _2\in {\mathcal {L}}_{g}(X)} \sum _{k_1,k_2\ge 1} \frac{ H_L(k_1\ell _1) H_L(k_2\ell _2) }{k_1 k_2 } U_T(k_1\ell _1,k_2\ell _2). \end{aligned}$$

Below we will be able to deduce Theorem 1.1 from the following two results: Proposition 2.2 proved at the end of Sect. 3, asserting that the random variables ${\mathcal {V}}_{T,L}(X)$ (indexed by *g*) converge, as $g\rightarrow \infty$, in distribution, to ${\mathcal {V}}_{T,L}^{\infty }$, and Theorem 2.3, proved along Sect. 4, asserting that, in the regime of Theorem 1.1, $\mathcal {V}_{T,L}^{\infty }$ converge, in distribution, to the constant $\Sigma ^2_{\textrm{GOE}}(f)$.

### Proposition 2.2

For every $T,L>0$, the random variables ${\mathcal {V}}_{T,L}={\mathcal {V}}_{T,L}(X)$, with $X\in {\mathcal {M}}_g$ random uniform w.r.t. the WP measure, converge, in distribution as $g\rightarrow \infty$, to the random variable ${\mathcal {V}}_{T,L}^{\infty }$.

### Theorem 2.3

One has

$$\begin{aligned} \lim _{\begin{array}{c} L,T\rightarrow \infty \\ L=o(\log T) \end{array}} {{\mathbb {E}}}_{\operatorname {Pois}}\left[ \left| {\mathcal {V}}_{T,L}^\infty -\Sigma ^2_{\textrm{GOE}}(f) \right| \right] = 0. \end{aligned}$$

Theorem 2.3 with Markov’s inequality implies that the probability that $\operatorname {Var}_T(S_{L,\tau } ^2)$ is far from $\Sigma ^2_{\textrm{GOE}}(f)$ vanishes: for every $\epsilon >0$,

2.9 $$\begin{aligned} \lim _{\begin{array}{c} L,T\rightarrow \infty \\ L=o(\log T) \end{array}}\operatorname {Prob}_{\operatorname {Pois}} \left( \left| {\mathcal {V}}_{T,L}^\infty -\Sigma ^2_{\textrm{GOE}}(f) \right| >\epsilon \right) =0. \end{aligned}$$

## Background on Point Processes

Let ${\mathcal {N}}$ be the space of measures $\mu$ on ${{\mathbb {R}}}_{\ge 0}$ s.t. $\mu (\{0\})=0$, for every $t\ge 0$, $\mu (t)\in {{\mathbb {Z}}}_{\ge 0}$, where $F_{\mu }(t):=\mu ((0,t])$ is the cumulative distribution function (CDF) associated with $\mu$, that is assumed to be right-continuous at 0. A measure $\mu \in {\mathcal {N}}$ has a representation

3.1 $$\begin{aligned} \mu =\sum \limits _{j=1}^{k}\delta _{\xi _{j}}, \end{aligned}$$

where $0\le k\le +\infty$, and $0<\xi _{1}\le \xi _{2}\le \ldots$, and sometimes it is convenient to regard $\mu$ as a discrete multi-set

3.2 $$\begin{aligned} \mu =\{\xi _{j}\}_{j\ge 1}\subseteq {{\mathbb {R}}}. \end{aligned}$$

Given a sequence $\{\mu ^{n}\}\subseteq {\mathcal {N}}$ and $\mu \in {\mathcal {N}}$, we say that $\mu ^{n}$ *vaguely converges* to $\mu$, denoted $\mu ^{n}\rightarrow \mu$, if for all $t\in {{\mathbb {R}}}_{\ge 0}$ so that $F_{\mu }(\cdot )$ is continuous at *t*,

3.3 $$\begin{aligned} \lim \limits _{n\rightarrow \infty }F_{\mu ^{n}}(t)=F_{\mu }(t), \end{aligned}$$

with $F_{\mu ^{n}}$ and $F_{\mu }$ the CDF of $\mu ^{n}$ and $\mu$ respectively. Equivalently, for every nonnegative continuous test function of compact support $f:{{\mathbb {R}}}_{\ge 0}\rightarrow {{\mathbb {R}}}$, one has

$$\begin{aligned} \int \limits _{0}^{\infty }f(x)\mu ^{n}(dx) \rightarrow \int \limits _{0}^{\infty }f(x)\mu (dx). \end{aligned}$$

A point process (in a somewhat restrictive sense, sufficient for our purposes.^3^) is a *random* element $\mu \in {\mathcal {N}}$ (w.r.t. the vague topology on ${\mathcal {N}}$, generating the Borel $\sigma$-algebra on ${\mathcal {N}}$). (Equivalently, it is a probability measure on ${\mathcal {N}}$.) We further assume that the points $\{\xi _{j}\}_{j\ge 1}$ are a.s. *distinct* (valid, provided that the corresponding point process is *simple*).

### Definition 3.1

 

i. A point process $\eta$ is Poisson with intensity measure $\nu$ on ${{\mathbb {R}}}_{>0}$, if for every Borel set $B\subseteq {{\mathbb {R}}}$, the distribution of $\eta (B)$ is Poisson with parameter $\nu (B)$, and the random variables $\eta (B_{1}),\ldots ,\eta (B_{k})$ are independent for every $k\ge 2$, and any choice of *disjoint* Borel sets $B_{1},\ldots , B_{k}$.
ii. A sequence of point processes $\eta _n$ on ${{\mathbb {R}}}_{>0}$ is said to converge (in distribution) to a point process $\eta$ (written $\eta _n \overset{d}{\rightarrow }\eta$) if the sequence of random vectors $(\eta _{n}(B_1),\dots , \eta _{n}(B_k))$ converges in distribution to the random vector $(\eta (B_1),\dots , \eta (B_k))$ for all $k\ge 1$ and Borel sets $B_i$ with boundaries satisfying $\mathbf \eta (\partial B_i)=0$ almost surely for all *i*.

Alternatively, a sequence $\eta _{n}$ of point processes, and a point process $\eta$ could also be seen as a ${\mathcal {N}}$-valued *random variable*, and, as such, the convergence, in distribution, of $\eta _{n}$ to $\eta$ makes sense. It is a deep and powerful result [3, Theorem 23 on p. 521] in probability theory, that the convergence of $\eta _n$ to $\eta$ in the sense of Definition 3.1(ii.) implies the convergence in distribution of the ${\mathcal {N}}$-valued random variables $\eta _n$ to $\eta$. That would allow us to employ the continuous mapping theorem, applicable, under certain conditions, in the context of the convergence in distribution of random variables.

For a measure $\mu \in {\mathcal {N}}$ as in (3.1) with $k\ge 1$ finite, and $1\le m\le k$, one defines [5, p. 28] the *m*’th *factorial measure* $\mu ^{(m)}$ of $\mu$ on ${{\mathbb {R}}}_{\ge 0}^{m}$ as

$$\begin{aligned} \mu ^{(m)} = \sum \limits _{i_{1},\ldots i_{m} \text { distinct}}\delta _{(\xi _{i_{1}},\ldots \xi _{i_{m}})}. \end{aligned}$$

Next, for a point process $\eta$ we define [5, Definition 4.9] the *m*’th factorial *moment* measure as

$$\begin{aligned} \alpha _{m}(C)=\alpha _{\eta ;m}(C):= {{\mathbb {E}}}\left[ \eta ^{(m)}(C)\right] \end{aligned}$$

for $C\subseteq {{\mathbb {R}}}_{\ge 0}^{m}$ measurable (Borel). Then the *m*’th factorial moment measure of a Poisson point process $\eta$ with intensity $\nu$ is [5, Corollary 4.10] $\nu ^{m}$, the *m*’th product measure

$$\begin{aligned} d\nu ^{m}(x_{1},\ldots ,x_{m})=d\nu (x_{1})\cdot \ldots d\nu (x_{m}) \end{aligned}$$

of $$\nu $$ on $${{\mathbb {R}}}_{\ge 0}^{m}$$.

In what follows, we use the identification (3.2) of a measure $\mu \in {\mathcal {N}}$ (or a random element of a point process $\eta$). Thus, the factorial moment measure of a *proper* point process $\eta$ (a class of point processes that includes any Poisson point process) computes the correlations of $\{\xi _{j}\}$ in the sense that for a measurable function $h:{{\mathbb {R}}}_{\ge 0}^{m}\rightarrow {{\mathbb {R}}}$,

3.4 $$\begin{aligned} {{\mathbb {E}}}\left[ \sum _{\begin{array}{c} (\xi _{1},\dots ,\,\xi _{m})\in \eta ^{m}\\ \text { distinct} \end{array}} h(\xi _{1},\dots ,\xi _{m}) \right] = \int \limits _{{{\mathbb {R}}}_{\ge 0}^{m}}h(\xi _{1},\ldots ,\xi _{m})d\alpha _{m}(\xi _{1},\dots ,\xi _{m}) , \end{aligned}$$

provided that the integral on the r.h.s. of (3.4) is convergent, see [5, Definition 4.9 and immediately after]. In particular, for $m=1$, this is Campbell’s formula [5, Proposition 2.7].

We conclude this section with the following observation. By comparing (2.5) to (2.8), we may express both as

$$\begin{aligned} {\mathcal {V}}_{T,L}(X) = \varphi _{T,L}({\mathcal {L}}_{g});\;\; {\mathcal {V}}_{T,L}^{\infty } = \varphi _{T,L}({\mathcal {L}}_{\infty }), \end{aligned}$$

with ${\mathcal {L}}_{g}$ and ${\mathcal {L}}_{\infty }$ (viewed as random point processes) as in Notation 2.1, and the functional $\varphi _{T,L}:{\mathcal {N}}\rightarrow {{\mathbb {R}}}$, defined as

3.5 $$\begin{aligned} \varphi _{T,L}(\mu ) := \left( \frac{2}{L} \right) ^2\sum _{\ell _1,\ell _2\in \mu } \sum _{k_1,k_2\ge 1} \frac{ H_L(k_1\ell _1) H_L(k_2\ell _2) }{k_1 k_2 } U_T(k_1\ell _1,k_2\ell _2), \end{aligned}$$

where the elements $\ell _{j}$ of $\mu$, taking the role of $\xi _{j}$, are reminiscent of the elements of the length spectrum ${\mathcal {L}}_{g}$. Appealing to the continuity and the compact support of $H_{L}(\cdot )$, this is easily seen to be continuous (with ${\mathcal {N}}$ equipped with the vague topology), see [12, Lemma 2.1].

### Proof of Proposition 2.2

We interpret the result of Mirzakhani–Petri [8, Theorem 4.1] as the convergence, as $g\rightarrow \infty$, of the point processes ${\mathcal {L}}_{g}$ to the Poisson point process ${\mathcal {L}}_{\infty }$ (Notation 2.1), in the sense of convergence of point processes, as in Definition 3.1(ii.). We recall that it implies that the random element ${\mathcal {L}}_{g}\in {\mathcal {N}}$ converges, in distribution, to ${\mathcal {L}}_{\infty }\in {\mathcal {N}}$, see the comments immediately after Definition 3.1. Hence, an application of the continuous mapping theorem, with the continuous functional $\varphi :{\mathcal {N}}\rightarrow {{\mathbb {R}}}$ as in (3.5), yields that, as $g\rightarrow \infty$, the random variables

$$\begin{aligned} {\mathcal {V}}_{T,L}(X) =\varphi ({\mathcal {L}}_{g}) \end{aligned}$$

converge, in distribution, to

$$\begin{aligned} {\mathcal {V}}_{T,L}^{\infty } = \varphi ({\mathcal {L}}_{\infty }), \end{aligned}$$

that is, the statement of Proposition 2.2. $\square$

## GOE Fluctuations for the Poisson Model: Proof of Theorem 2.3

We need to compute the expected value of $\left| {\mathcal {V}}_{T,L}^\infty -\Sigma ^2_{\textrm{GOE}}(f) \right|$ with ${\mathcal {V}}_{T,L}^\infty$ given by (2.5) with $\ell _1,\ell _2$ selected according to the Poisson point process. We write (2.5) according to whether $\ell _1=\ell _2$ (the diagonal) and $\ell _1\ne \ell _2$:

$$\begin{aligned} {\mathcal {V}}_{T,L}^\infty = \sum \limits _{\begin{array}{c} \ell _{1},\ell _{2}\in {\mathcal {L}}_{\infty }\\ \ell _1=\ell _2 \end{array}} + \sum _{\begin{array}{c} \ell _{1},\ell _{2}\in {\mathcal {L}}_{\infty }\\ \ell _1\ne \ell _2 \end{array}} = \operatorname {Diag}+ \operatorname {Off}\end{aligned}$$

and then we have

$$\begin{aligned} {{\mathbb {E}}}_{\operatorname {Pois}}\left[ \left| {\mathcal {V}}_{T,L}^\infty -\Sigma ^2_{\textrm{GOE}}(f) \right| \right] \le {{\mathbb {E}}}_{\operatorname {Pois}}\left[ \left| \operatorname {Diag}-\Sigma ^2_{\textrm{GOE}}(f) \right| \right] + {{\mathbb {E}}}_{\operatorname {Pois}}\left[ \left| \operatorname {Off}\right| \right] . \end{aligned}$$

We will show that both terms tend to zero as $L,T\rightarrow \infty$, $L=o(\log T)$.

### Bounding ${{\mathbb {E}}}_{\operatorname {Pois}}\left[ \left| \operatorname {Off}\right| \right]$

#### Lemma 4.1

There is some $C_{0}=C_0(f)>0$ so that

4.1 $$\begin{aligned} {{\mathbb {E}}}_{\operatorname {Pois}}\left[ \left| \operatorname {Off}\right| \right] \ll \frac{e^{2C_{0}L}}{TL}, \end{aligned}$$

the constant involved in the ‘$\ll$’-notation depending only on $f,\omega$.

#### Proof

We apply the correlation formula (3.4) with $m=2$, the function *h* given by

$$\begin{aligned} h(\xi _{1},\xi _{2}) = \frac{4}{L^{2}}\sum \limits _{k_{1},k_{2}\ge 1} \frac{|H_{L}(k_{1}\xi _{1})|\cdot |H_{L}(k_2\xi _2))|}{k_1 k_2} | U_T(k_{1}\xi _{1},k_{2}\xi _{2})|, \end{aligned}$$

and intensity $d\nu _{MP}(x) = 2\sinh ^2(x/2)/x \;dx$, to yield that

4.2 $$\begin{aligned} {{\mathbb {E}}}_{\operatorname {Pois}}\left[ \left| \operatorname {Off}\right| \right]\le &   {{\mathbb {E}}}_{\operatorname {Pois}}\left[ \sum _{\begin{array}{c} \ell _{1},\ell _{2}\in {\mathcal {L}}_{\infty }\\ \ell _1\ne \ell _2 \end{array}} h(\ell _{1},\ell _{2}) \right] \nonumber \\= &   \int \limits _{0}^{\infty }\int \limits _{0}^{\infty }h(x,y)d\nu _{MP}(x)d\nu _{MP}(y) \nonumber \\= &   \frac{4}{L^{2}}\sum \limits _{k_{1},k_{2}\ge 1}\int \limits _{0}^{\infty }\int \limits _{0}^{\infty } \frac{| H_{L}(k_{1}x)H_{L}(k_{2}y)|}{k_1k_2} |U_T(k_1x,k_2y)| d\nu _{MP}(x)d\nu _{MP}(y) \nonumber \\= &   \frac{16}{L^{2}}\sum \limits _{k_{1},k_{2}\ge 1}\int \limits _{0}^{\infty }\int \limits _{0}^{\infty } \left| {\widehat{f}}\left( \frac{k_{1}x}{L} \right) \cdot {\widehat{f}}\left( \frac{k_{2}y}{L} \right) \right| \cdot \frac{\sinh (x/2)^{2}\cdot \sinh (y/2)^{2}}{\sinh \left( \frac{k_{1}x}{2} \right) \cdot \sinh \left( \frac{k_{2}y}{2} \right) }\nonumber \\  &   \times \,|U_T(k_1x,k_2y)| dxdy \end{aligned}$$

by (2.6). In what follows we show that when $L=o(\log {T})$, this expression vanishes.

We note that ${\widehat{f}}$ being compactly supported forces that in the range of the integral one has $0< x\ll \frac{L}{k_{1}}$, so that $\sinh (x/2) \le e^{C_{0}\cdot L}$ for some $C_{0}>0$ depending only on $f,\omega$, and likewise $\sinh (y/2) \le e^{C_{0}\cdot L}$. Since for $k\ge 1$ and $t\in {{\mathbb {R}}}$

$$\begin{aligned} \frac{\sinh (t)}{\sinh (kt)} \le \frac{1}{k}, \end{aligned}$$

we have

$$\begin{aligned} \frac{\sinh (x/2)^{2}\cdot \sinh (y/2)^{2}}{\sinh \left( \frac{k_{1}x}{2} \right) \cdot \sinh \left( \frac{k_{2}y}{2} \right) } \le e^{2C_{0}\cdot L}\cdot \frac{1}{k_{1}k_{2}}. \end{aligned}$$

Given *x* in the domain of the integration (as above, $x\ll \frac{L}{k_{1}}$), the compact support of ${\widehat{\omega }}$ forces that *y* is contained in an interval of length $\frac{1}{k_{2}T}$ (from (2.7)). Therefore, taking into account the boundedness of both ${\widehat{f}}$ and ${\widehat{w}}$ (hence of $U_T$), we have

4.3 $$\begin{aligned} \begin{aligned} \frac{1}{L^{2}}\int \limits _{0}^{\infty }\int \limits _{0}^{\infty }&\left| {\widehat{f}}\left( \frac{k_{1}x}{L} \right) \cdot {\widehat{f}}\left( \frac{k_{2}y}{L} \right) \right| \frac{\sinh (x/2)^{2} \sinh (y/2)^{2}}{\sinh \left( \frac{k_{1}x}{2} \right) \sinh \left( \frac{k_{2}y}{2} \right) } |U_T(k_1x,k_2y)| dxdy \\&\ll \frac{1}{L^{2}}\cdot \frac{e^{2C_{0}L}}{k_{1}k_{2}}\cdot \frac{1}{Tk_{2}}\cdot \frac{L}{k_{1}} = \frac{e^{2C_{0}L}}{TL}\cdot \frac{1}{k_{1}^{2}k_{2}^{2}}. \end{aligned} \end{aligned}$$

The bound (4.1) of Lemma 4.1 finally follows upon summing up (4.3) for $k_{2}, k_{1}\ge 1$ and substituting it into (4.2). $\square$

### Bounding the Diagonal Term

We now want to bound the term ${{\mathbb {E}}}_{\operatorname {Pois}}\left[ \left| \operatorname {Diag}-\Sigma ^2_{\textrm{GOE}}(f) \right| \right]$. Recall

$$\begin{aligned} \operatorname {Diag}= \frac{4}{L^2}\sum \limits _{\ell \in {\mathcal {L}}_{\infty }} \sum _{k_1,k_2\ge 1} \frac{ H_L(k_1\ell ) H_L(k_2\ell ) }{k_1 k_2 } U_T(k_1\ell ,k_2\ell ) \end{aligned}$$

We separate out the contribution of the pair $(k_1,k_2)=(1,1)$ and the rest and use

4.4 $$\begin{aligned}  &   {{\mathbb {E}}}_{\operatorname {Pois}}\left[ \left| \operatorname {Diag}-\Sigma ^2_{\textrm{GOE}}(f) \right| \right] \nonumber \\  &   \quad \le {{\mathbb {E}}}_{\operatorname {Pois}}\left[ \left| \frac{4}{L^2}\sum _x H_L(x)^2 U_T(x,x) - \Sigma ^2_{\textrm{GOE}}(f) \right| \right] \nonumber \\  &   \quad \quad + \frac{4}{L^2} {{\mathbb {E}}}_{\operatorname {Pois}}\left[ \sum \limits _{\ell \in {\mathcal {L}}_{\infty }} \sum _{k_1+k_2\ge 3} \frac{| H_L(k_1\ell ) H_L(k_2\ell )| }{k_1 k_2 } |U_T\left( k_1\ell ,k_2\ell \right) | \right] . \end{aligned}$$

#### The Sum $k_1+k_2\ge 3$

We set

$$\begin{aligned} D(k_1,k_2):= \frac{4}{L^2} {{\mathbb {E}}}_{\operatorname {Pois}}\left[ \sum \limits _{\ell \in {\mathcal {L}}_{\infty }} \frac{| H_L(k_1\ell ) H_L(k_2\ell )| }{k_1 k_2 } |U_T\left( k_1\ell ,k_2\ell \right) | \right] \end{aligned}$$

and want to show that

$$\begin{aligned} \sum _{k_1+k_2\ge 3} D(k_1,k_2) \ll \frac{\log L}{L^2}. \end{aligned}$$

We apply Campbell’s formula ((3.4) with $m=1$)

4.5 $$\begin{aligned} {{\mathbb {E}}}_{\operatorname {Pois}}\left[ \sum \limits _{\ell \in {\mathcal {L}}_{\infty }} h(\ell ) \right] =\int _0^\infty h(x) d\nu _{MP}(x) \end{aligned}$$

with $h(x) = \frac{| H_L(k_1x) H_L(k_2x)| }{k_1 k_2 } |U_T\left( k_1x,k_2x \right) |$ to find

$$\begin{aligned} \begin{aligned} D(k_1,k_2)&= \frac{4}{L^2}\int _0^\infty \frac{| H_L(k_1x) H_L(k_2x)| }{k_1 k_2 } |U_T\left( k_1x,k_2x \right) | d\nu _{MP}(x) \\&\ll \int _0^{C_0 L} \frac{\sinh ^2(Lx/2)}{\sinh (Lk_1x/2)\sinh (Lk_2x/2)} x dx \end{aligned} \end{aligned}$$

on using boundedness of $U_T$ and ${\widehat{f}}$ being supported in $[-C_0,C_0]$. It is easy to show (see the proof of [11, Lemma 5.2]) that this last integral is bounded by

$$\begin{aligned} \ll \min \left( \frac{1}{L^2(k_1+k_2-2)^2}, \frac{1}{k_1k_2^3} \right) \end{aligned}$$

(assuming $k_2\ge k_1$) and from this to show that

$$\begin{aligned} \sum _{k_1+k_2\ge 3} D(k_1,k_2) \ll \frac{\log L}{L^2} \end{aligned}$$

which is our claim.

#### The Term $k_1=k_2=1$

We are left with showing that the first term on the RHS of (4.4) tends to zero, and using Cauchy–Schwartz it suffices to bound the second moment

$$\begin{aligned} \lim _{L\rightarrow \infty } {{\mathbb {E}}}_{\operatorname {Pois}}\left[ \left| \frac{4}{L^2}\sum \limits _{\ell \in {\mathcal {L}}_{\infty }} H_L(\ell )^2 U_T(\ell ,\ell ) - \Sigma ^2_{\textrm{GOE}}(f) \right| ^2 \right] = 0 \end{aligned}$$

for $T\ge 1$. Squaring out, we want to show

$$\begin{aligned}  &   {{\mathbb {E}}}_{\operatorname {Pois}}\left[ \left( \frac{4}{L^2} \sum \limits _{\ell \in {\mathcal {L}}_{\infty }} H_L(\ell )^2U_T(\ell ,\ell ) \right) ^2 \right] \\  &   \quad \quad -2\Sigma ^2_{\textrm{GOE}}(f) {{\mathbb {E}}}_{\operatorname {Pois}}\left[ \frac{4}{L^2}\sum \limits _{\ell \in {\mathcal {L}}_{\infty }} H_L(\ell )^2U_T(\ell ,\ell ) \right] \rightarrow -(\Sigma ^2_{\textrm{GOE}}(f))^2. \end{aligned}$$

We have

4.6 $$\begin{aligned} {{\mathbb {E}}}_{\operatorname {Pois}}\left[ \frac{4}{L^2} \sum \limits _{\ell \in {\mathcal {L}}_{\infty }} H_L(\ell )^2 U_T(\ell ,\ell )\right] = \Sigma ^2_{\textrm{GOE}}(f) +O\left( \frac{1}{TL} \right) . \end{aligned}$$

Indeed, by Campbell’s formula (4.5),

$$\begin{aligned}  &   {{\mathbb {E}}}_{\operatorname {Pois}}\left[ \frac{4}{L^2} \sum \limits _{\ell \in {\mathcal {L}}_{\infty }} H_L(\ell )^2 U_T(\ell ,\ell )\right] \\  &   \quad =\frac{4}{L^2} \int _0^\infty H_L(x)^2 U_T(x,x) d\nu _{MP}(x) \\  &   \quad = \frac{4}{L^2} \int _0^\infty \frac{x^2{\widehat{f}}\left( \frac{x}{L} \right) }{\sinh ^2(x/2)} \frac{1}{2}\left( 1+{\widehat{w}}(2Tx)-2{\widehat{w}}(Tx)^2 \right) 2\frac{\sinh ^2(x/2)}{x}dx \\  &   \quad = 4\int _0^\infty {\widehat{f}}(y)^2 y dy+ 4\int _0^\infty {\widehat{f}}(y)^2 y \left( {\widehat{w}}(2TL y) - 2{\widehat{w}}(TLy)^2\right) dy, \end{aligned}$$

using

$$\begin{aligned} U_T(x,x) = \frac{1}{2} + \frac{1}{2} {\widehat{w}}(2Tx) -{\widehat{w}}(Tx)^2. \end{aligned}$$

The first term is $2\int _{-\infty }^\infty {\widehat{f}}(y)^2|y|dy = \Sigma ^2_{\textrm{GOE}}(f)$. The second term is treated by observing that since $\operatorname {Supp}{\widehat{w}}\subseteq [-1,1]$, the integral is bounded by

$$\begin{aligned} \int _0^\infty {\widehat{f}}(y)^2 y \left( |{\widehat{w}}(2TL y)| + 2|{\widehat{w}}(TLy)^2| \right) dy\ll \int _0^{1/2TL} {\widehat{f}}(y)^2 ydy \ll \frac{1}{TL}, \end{aligned}$$

hence we obtain (4.6)

Therefore, it suffices to show

$$\begin{aligned} {{\mathbb {E}}}_{\operatorname {Pois}}\left[ \left( \frac{4}{L^2}\sum \limits _{\ell \in {\mathcal {L}}_{\infty }} H_L(\ell )^2 U_T(\ell ,\ell )\right) ^2 \right] \rightarrow (\Sigma ^2_{\textrm{GOE}}(f))^2. \end{aligned}$$

Again square out, obtain a diagonal sum and an off-diagonal sum.

We bound the diagonal sum using Campbell’s formula (4.5)

$$\begin{aligned} {{\mathbb {E}}}_{\operatorname {Pois}}\left[ \frac{16}{L^4} \sum \limits _{\ell \in {\mathcal {L}}_{\infty }} H_L(\ell )^4 U_T(\ell ,\ell )^2 \right]= &   \frac{16}{L^4}\int _0^\infty H_L(x)^4 U_T(x,x)^2 d\nu _{MP}(x) \\= &   \frac{16}{L^4} \int _0^\infty \frac{x^4 {\widehat{f}}\left( \frac{x}{L} \right) ^4}{\sinh ^4(x/2)} \frac{2\sinh ^2(x/2)}{x} U_T(x,x)^2 dx \\\ll &   \frac{1}{L^4} \int _0^{cL} \frac{ x^3}{\sinh ^2(x/2)}dx \ll \frac{1}{L^4}. \end{aligned}$$

We evaluate the off-diagonal term using the correlation formula (3.4) with $m=2$ for the Poisson process

$$\begin{aligned}  &   {{\mathbb {E}}}_{\operatorname {Pois}}\left[ \frac{16}{L^4} \sum _{\begin{array}{c} \ell _{1},\ell _{2}\in {\mathcal {L}}_{\infty }\\ \ell _{1}\ne \ell _{2} \end{array}} H_L(\ell _{1})^2 U_T(\ell _{1},\ell _{1}) H_L(\ell _{2})^2 U_T(\ell _{2},\ell _{2}) \right] \\  &   \quad =\frac{16}{L^4}\iint H_L(x)^2 U_T(x,x) H_L(y)^2U_T(y,y) d\nu _{MP}(x)d\nu _{MP}(y) \\  &   \quad = \left( \frac{4}{L^2}\int _0^\infty H_L(x)^2 U_T(x,x) d\nu _{MP}(x) \right) ^2 = \left( \Sigma ^2_{\textrm{GOE}}(f) \right) ^2 + O\left( \frac{1}{TL}\right) \end{aligned}$$

by (4.6), so we are done. $\square$

## Almost Sure GOE Fluctuations: Concluding the Proof of Theorem 1.1

### Proof

Let $\epsilon >0$ be given, and let us consider for a moment the random variables ${\mathcal {V}}_{T,L}={\mathcal {V}}_{T,L}(X)$ and ${\mathcal {V}}_{T,L}^{\infty }$ for sufficiently large, but fixed *T*, *L* within the allowed range $L=o(\log {T})$. It would follow that, as $g\rightarrow \infty$,

5.1 $$\begin{aligned} \operatorname {Prob}\left( \left| {\mathcal {V}}_{T,L}- \Sigma _{GOE}^{2}(f)\right|>\epsilon \right) \rightarrow \operatorname {Prob}\left( \left| {\mathcal {V}}_{T,L}^{\infty }- \Sigma _{GOE}^{2}(f)\right| >\epsilon \right) , \end{aligned}$$

provided that $\Sigma _{GOE}^{2}(f)\pm \epsilon$ are not atoms of the distribution of ${\mathcal {V}}_{T,L}^{\infty }$. Otherwise, apply (5.1) with

$$\begin{aligned} \epsilon /2<\epsilon '=\epsilon '(T,L)<\epsilon \end{aligned}$$

so that, for the given *T*, *L*, the distribution of ${\mathcal {V}}_{T,L}^{\infty }$ does not have an atom at $\Sigma _{GOE}^{2}(f)\pm \epsilon '$, and use that, for sufficiently large admissible *T*, *L*, the probability of

$$\begin{aligned} \left\{ \left| {\mathcal {V}}_{T,L}^{\infty }- \Sigma _{GOE}^{2}(f)\right|>\epsilon '\right\} \subseteq \left\{ \left| {\mathcal {V}}_{T,L}^{\infty }- \Sigma _{GOE}^{2}(f)\right| >\frac{\epsilon }{2}\right\} \end{aligned}$$

is arbitrarily small, by (2.9), a corollary from Theorem 2.3. Finally, the double limit statement (1.5) follows, as

$$\begin{aligned} \begin{aligned}&\operatorname {Prob}\left( \left| {\mathcal {V}}_{T,L}- \Sigma _{GOE}^{2}(f)\right|>\epsilon \right) \le \operatorname {Prob}\left( \left| {\mathcal {V}}_{T,L}- \Sigma _{GOE}^{2}(f)\right|>\epsilon ' \right) \\&\quad = \operatorname {Prob}\left( \left| {\mathcal {V}}_{T,L}^{\infty }- \Sigma _{GOE}^{2}(f)\right|>\epsilon ' \right) \\&\quad \quad + \bigg (\operatorname {Prob}\left( \left| {\mathcal {V}}_{T,L}- \Sigma _{GOE}^{2}(f)\right|>\epsilon ' \right) -\operatorname {Prob}\left( \left| {\mathcal {V}}_{T,L}^{\infty }- \Sigma _{GOE}^{2}(f)\right|>\epsilon ' \right) \bigg ) \\&\quad \le \operatorname {Prob}\left( \left| {\mathcal {V}}_{T,L}^{\infty }- \Sigma _{GOE}^{2}(f)\right|>\frac{\epsilon }{2} \right) \\&\qquad + \bigg (\operatorname {Prob}\left( \left| {\mathcal {V}}_{T,L}- \Sigma _{GOE}^{2}(f)\right|>\epsilon ' \right) -\operatorname {Prob}\left( \left| {\mathcal {V}}_{T,L}^{\infty }- \Sigma _{GOE}^{2}(f)\right| >\epsilon ' \right) \bigg ) , \end{aligned} \end{aligned}$$

with the first term small by (2.9), and the difference tends to zero as $g\rightarrow \infty$ since $\Sigma _{GOE}^{2}(f)\pm \epsilon '$ is not an atom of ${\mathcal {V}}_{T,L}^{\infty }$. $\square$

## Footnotes

1 Arithmetic surfaces are exceptional, see [2, 6].

2 Genus $g=2$ requires a modification due to the presence of the hyperelliptic involution.

3 In particular, we assume that all our processes are *proper*.
